# Three-Dimensional-Printed Isoniazid Chewable Gels for On-Demand Latent Tuberculosis Treatment in Children

**DOI:** 10.3390/pharmaceutics17050658

**Published:** 2025-05-17

**Authors:** Amanda de O. E. Moreira, Lêda Maria S. Azevedo Neta, Márcia Pietroluongo, Ana Paula dos S. Matos, Beatriz B. Correa, Beatriz H. Ortiz, André da S. Guimarães, Marcio Nele, Carollyne M. Santos, Ana Elizabeth C. Fai, Maria Helena Gonçalves, Flávio M. Shimizu, Monique S. Dos Santos, Rosemberg B. Moure, Diogo D. Nascimento, André Luis de A. Guimarães, Saint Clair dos S. G. Junior, Alessandra L. Vicosa, Lucio M. Cabral

**Affiliations:** 1 Laboratory of Pharmaceutical Industrial Technology (LabTIF), Faculty of Pharmacy, Federal University of Rio de Janeiro, Rio de Janeiro 21941-902, Brazil; amandaoemfarma@gmail.com; 2 Laboratory of Experimental Pharmacotechnics, Department of Galenic Innovation, Oswaldo Cruz Foundation (FIOCRUZ), Farmanguinhos, Rio de Janeiro 22775-903, Brazil; leda.neta@fiocruz.br (L.M.S.A.N.); marcia.pietroluongo@fiocruz.br (M.P.); paula.matos@fiocruz.br (A.P.d.S.M.); beatriz.correa@fiocruz.br (B.B.C.); beatrizhecht11@gmail.com (B.H.O.); 3 Colloid Engineering Laboratory, Alberto Luiz Coimbra Institute of Graduate Studies and Research in Engineering (COPPE), Federal University of Rio de Janeiro, Rio de Janeiro 21941-598, Brazil; asguimaraes@peq.coppe.ufrj.br (A.d.S.G.); nele@eq.ufrj.br (M.N.); 4 Food and Nutrition Graduate Program, Federal University of Rio de Janeiro State, Rio de Janeiro 22290-250, Brazil; carolmaragoni@hotmail.com; 5 Laboratory of Multidisciplinary Practices for Sustainability (LAMPS), Institute of Nutrition, Rio de Janeiro State University (UERJ), Rio de Janeiro 20550-170, Brazil; bethfai@icloud.com; 6 Gleb Wataghin Institute of Physics, State University of Campinas, Campinas 13083-859, Brazil; mariahelenags10@gmail.com (M.H.G.); fshimizu@unicamp.br (F.M.S.); 7 Analytical Development and Validation Laboratory, Fundação Oswaldo Cruz (FIOCRUZ), Farmanguinhos, Manguinhos, Rio de Janeiro 22775-903, Brazil; monique.santos@fiocruz.br (M.S.D.S.); rosemberg.moure@fiocruz.br (R.B.M.); diogo.nascimento@fiocruz.br (D.D.N.); 8 Department of Natural Products and Food, Federal University of Rio de Janeiro, Rio de Janeiro 21941-902, Brazil; andreluis.guimaraes@gmail.com; 9 Clinical Research Unit, Fernandes Figueira National Institute of Women’s, Children’s and Adolescents’ Health (IFF), Oswaldo Cruz Foundation (FIOCRUZ), Flamengo, Rio de Janeiro 22250-020, Brazil; saint.junior@fiocruz.br

**Keywords:** tuberculosis, isoniazid, pediatric medicines, chewable gels, 3D printing, semi-solid extrusion, pharmaceutical manufacturing, rheology

## Abstract

**Background/Objectives**: Pediatric drug administration is hindered by difficulties in swallowing conventional medications and the unpalatable taste of many drugs. Among diseases highlighting the need for improved pediatric delivery, tuberculosis (TB) stands out. One form of the disease is latent TB infection (LTBI), which is concerning in children. Effective LTBI treatment is crucial for prevention, with isoniazid (INH) widely used for its proven efficacy and safety. This study aims to develop innovative 3D-printed chewable gels containing INH for LTBI treatment. **Methods**: The gels were formulated using gelatin and carrageenan gum, sugar-free sweeteners, and flavoring. Two batches were prepared, and using 3D printing (3DP) with a semi-solid extrusion (SSE) module, chewable gels were produced. Rheological properties were measured to assess the feasibility of 3DP-SSE, evaluating the structural integrity and adequate fluidity of the formulation. The 3D-printed chewable gels were evaluated by visual, mass, and dimensional characteristics. In addition, the water activity, texture profile, INH and degradation product content, in vitro release, and taste-masking were investigated. **Results**: The optimized formulation maintained suitable rheological properties for 3DP-SSE, demonstrating consistent weight, dimensions, and stability after the process. The texture achieved a balance between printing parameters and shape maintenance, and the INH presented an immediate-release profile (>85% within 30 min). The chewable gels showed an improvement in palatability compared to conventional INH tablets. **Conclusions**: This innovative approach offers a promising solution for pediatric LTBI treatment, as it improves efficacy, medication acceptability, and on-demand access.

## 1. Introduction

Children frequently face challenges in swallowing traditional medications such as tablets and capsules. Additionaly, many find the taste unpalatable, making pediatric drug delivery a challenge [1]. Factors such as fear, discomfort, and lack of parental knowledge about proper administration also contribute to treatment refusal and dosing errors [2]. These challenges are further compounded by gaps in pediatric drug research. Despite initiatives like the “Best Pharmaceuticals for Children Act”, pediatric drug research remains insufficient, with many drugs lacking proper usage guidelines and off-label use being common [3]. As a result, there is increasing recognition of the need for customized medicines and novel drug delivery systems tailored to the unique needs of children [4,5,6,7]. Among the diseases that particularly highlight the need for improved pediatric drug delivery, tuberculosis (TB) stands out.

TB is a bacterial infection recognized as a global epidemic by the World Health Organization (WHO) in 1993. Currently, this disease is the leading cause of death from a single infectious agent in the world, and is one of the greatest challenges to public health [8]. Approximately one-third of the world’s population is latently infected with *Mycobacterium tuberculosis*. In children, one variety of the disease is latent TB infection (LTBI), which is particularly concerning due to the significant risk of progression to active disease [9,10]. Effective treatment of LTBI is essential to prevent disease progression. The WHO recommends several regimens, including 6 or 9 months of daily isoniazid (INH), 3 months of rifapentine in combination with weekly INH, or 3 months of daily INH in combination with rifampicin. Additionally, alternative regimens are suggested, such as 1 month of rifapentine in combination with daily INH, or 4 months of daily rifampicin, although these are supported by evidence of lower certainty [11].

INH monotherapy remains a relevant strategy for the treatment of LTBI in children, particularly in light of concerns regarding combined regimens. Although the regimen of 3 months of daily INH in combination with rifampicin has been adopted due to its shorter duration and favorable adherence, studies have reported a significant increase in serious adverse events in pediatric patients, including anaphylaxis and the need to discontinue treatment [12]. Furthermore, pharmacokinetic evidence indicates that the use of fixed-dose combination formulations containing INH may lead to overexposure to the active pharmaceutical ingredient (API), especially in young children, thereby increasing the risk of toxicity [13]. In this context, INH, when formulated as a single-drug pharmaceutical product, offers better dose control and greater flexibility in the therapeutic regimen to be adopted. Identifying and adequately treating LTBI is crucial to reduce the global TB burden [11].

To address these challenges, the WHO launched the End TB Strategy in 2015, aiming to reduce the number of cases by 80% by 2030 [14]. One of the pillars of this strategy is the intensification of research and innovation to develop new tools, interventions, and therapeutic strategies [15]. In this scenario, 3D printing (3DP) emerges as a promising technology that aligns with the need for innovation in pediatric and TB treatments, with the main advantage being the ability to safely personalize the dosage [16,17,18,19]. In 2015, the first 3D-printed medicine, Spritam, was approved by the Food and Drug Administration (FDA). In 2019, the first pilot clinical study on the use of pharmaceutical 3DP in pediatrics in a hospital setting was published [16,20]. Thus, compared to classical pharmaceutical techniques, the 3DP of medicines is innovative and disruptive compared to classical pharmaceutical techniques, revolutionizing the prototyping of new pediatric medications [21,22]. This technology facilitates on-demand manufacturing, making it particularly suitable for small-scale pharmaceutical production for neglected populations and diseases, such as children and ILTB. Additionally, 3DP promotes a circular economy by reducing waste, allowing feed material repurposing and generating less production residue [23,24,25].

Among the various 3DP methods, in semi-solid extrussion (SSE), 3DP is used to fabricate a semi-solid material containing the drug, extruded layer by layer in a syringe-based 3D printer, allowing the fabrication of products with the desired characteristics of shape, size, texture, and flavor [26,27]. To ensure adequate printing performance and final drug quality, rigorous control of the intermediate product properties in terms of rheology, extrudability, and uniformity is essential, as these factors directly influence process efficiency [26]. Maintaining control over these intermediate product properties is crucial to achieve optimal processing and reproducibility of 3D-printed medications [28,29].

A promising oral dosage form for pediatric patient adherence is chewable gels produced by SSE, due to their soft texture and good palatability. These gels are produced through the gelation of hydrocolloids and formulated with gelling agents, sweeteners, flavors, and occasionally dyes [30]. The preparation of the intermediate product involves critical steps such as mixing, heating, and syringe filling [29,31]. Compared to traditional dosage forms, chewable gels have particularities in characterization, whose complexity is amplified by 3DP production, requiring comprehensive analysis to ensure reliable production with consistent quality and desired attributes. In this context, this work aims to develop and characterize 3D-printed chewable gels containing the anti-tuberculosis drug INH for pediatric LTBI treatment.

## 2. Materials and Methods

### 2.1. Materials

Farmanguinhos/Fiocruz kindly donated INH from AMSAL CHEM™, Gujarat, India. Carrageenan gum was kindly donated by DuPont Nutriton (Rockland, PA, USA). Gelatin was kindly donated by Gelita (São Paulo, Brazil). Xylitol and maltitol syrup were kindly donated by Roquette (Lestrem, France). Strawberry flavor was kindly donated by Givaudan (Jaguaré, Brazil). All the solvents obtained were of analytical grade.

### 2.2. Preparation of the Intermediate Product

The selection of excipients was carried out based on a review of the scientific literature on the topic of chewable gel production. After that, the formulation was prepared in a closed system to minimize water loss and ensure uniform heating. The concentrations of the ingredients were selected to enhance the solid content of the final product. The composition of both the placebo and the INH-containing formulations is detailed in Table 1.

The batches were produced in a jacketed reactor with mechanical stirring (IKA, Staufen, Germany) and an anchor-type impeller. A condenser ensured water reflux, and the reactor’s heating bath (Thermo Scientific Phoenix II, Phoenix, AZ, USA) was set to 100 °C, maintaining the formulation at 90 °C. Purified water was added to the reactor. A physical mixture of xylitol 1, carrageenan, and gelatin was then added, and polymer hydration occurred with intermittent stirring. Meanwhile, the syrup was pre-heated at 80 °C. After polymer hydration, the syrup was added to the formulation under controlled stirring to prevent bubbles. Next, xylitol 2 and INH were incorporated under stirring, followed by the addition of strawberry flavor. The solids content was verified using a digital bench refractometer (IONLAB. MSDR-D, Xangai, China). While warm, the prepared solution was poured into syringes, and sealed with Parafilm after cooling to prevent contamination or water loss. Two INH batches, named 1 and 2, were prepared for reproducibility testing.

### 2.3. Rheological Analysis of the Intermediate Products

Rheological characterization was performed to evaluate the flow and structural properties of the intermediate products. The mechanical properties are essential for the suitability of a material for 3DP [28,29,33]. Each sample was analyzed only once to evaluate the behavior of the formulation. The tests were conducted using a Discovery HR-3 controlled stress rheometer (TA Instruments, New Castle, DE, USA) equipped with a 60 mm cross-hatched plate–plate geometry, a Peltier lower plate with temperature control, and a 1 mm gap. Approximately 2 g of each sample was loaded in a solid state and melted directly on the heated lower plate at 90 °C for 5 min. A sample cover apparatus was used to minimize evaporation during testing. Data analysis was performed with Trios Rheology Software V5.1.1.46572 (TA Instruments^®^, New Castle, DE, USA).

To investigate the rheological properties, unidirectional and oscillatory methods were applied. In the unidirectional tests, the protocol consisted of a temperature ramp from 90 to 40 °C at a cooling rate of 1.0 °C·min^−1^ and a shear rate of 1.0 s^−1^. This step was followed by a shear rate sweep ranging from 0.01 to 100 s^−1^ at a fixed temperature. The oscillatory tests consisted of strain amplitude sweeps to determine the linear viscoelastic range (LVR), covering strain amplitudes from 0.01 to 4000% at a frequency of 1 Hz. The yield stress was defined as the maximum stress amplitude value during the strain amplitude sweep. Elastic recovery tests simulated printing conditions, combining shear rates of 100 s^−1^ for 1 min with subsequent oscillation at 1% strain amplitude in the LVR for 5 min. The latter test assessed structural recovery under cyclic deformation.

To simulate the 3D printing process, samples were submitted to a temperature ramp from 90 to 70 °C at 2.0 °C·min^−1^, shear rate application, and a final oscillatory temperature ramp from 70 to 25 °C at 5 °C·min^−1^ (1% strain amplitude, 1 Hz frequency). This approach mimicked layer deposition and cooling to evaluate post-printing structural stability.

### 2.4. pH Determination of the Intermediate Product

The pH determination was conducted using the pH 791 method of the United States Pharmacopeia (USP). Herein, 15 g of each intermediate product was sliced into pieces and transferred to a beaker containing 15 mL of boiling water (80–90 °C). The beaker was then sealed and placed in a water bath with stirring at 55 °C for 30 min. After dissolution, the sample was cooled to 30 °C and the pH was measured (S20 SevenEasy Mettler Toledo, Greifensee, Zwitserland) [34].

### 2.5. FTIR Spectroscopy Characterization

FTIR spectroscopy was performed in ATR mode, using an IRTracer-100 (Shimadzu™, Kyoto, Japan) in the range of 500–4000 cm^−1^, to analyze the intermediates, aiming to ensure the identity, integrity, and compatibility of the materials used. The spectra resolution was defined at 4 cm^−1^, with 32 scan accumulations for each sample [35].

### 2.6. Differential Scanning Calorimetry (DSC) Analysis

Samples of pure INH, placebo intermediate products, and intermediate products containing INH were thermally analyzed using DSC with a sample mass of ~2 mg, in order to identify insolubilized materials. Thermograms were obtained using a DSC60 Differential Scanning Calorimeter (Shimadzu^®^, Kyoto, Japan), under a nitrogen gas flow of 50 mL·min^−1^. The samples were crimped in an aluminum pan and heated at 10 °C·min^−1^ from 0 to 250 °C. In a second trial, two heating cycles, from 30 to 200 °C, were conducted with a heating and cooling rate of 10 °C min^−1^. As a reference, a thermogram was obtained with an empty aluminum pan.

### 2.7. Preparation of 3D-Printed Chewable Gels

The 3DP process was planned using a virtual 3D model to achieve a target weight of 1 g. The model was designed in the free CAD software (https://www.tinkercad.com/ accessed on 12 November 2024) Tinkercad from Autodesk, Inc., San Francisco, CA, USA and converted to an STL file format. This file was then loaded into the free slicing software Repetier Host V2.2.4. The slicing software divided the model into layers, generating a G-code file for layer-by-layer printing. The printing step was conducted with an M3DIMAKER pharmaceutical 3D printer (FabRX, London, UK) equipped with an SSE head, using a 20 mL syringe and a 1.2 mm plastic nozzle.

Initial tests involved printing one or two chewable gel units, while adjusting variables such as the layer height, wall thickness, fill density, fill pattern, printing speed, and temperature. Z displacement and extrusion parameters were optimized to ensure product quality. Five minutes were allowed for drying after printing, before handling the chewable gels (the demonstration video can be found in Supplementary Video S1—Demonstration of the chewable gel printing process). Once optimized (Table 2), 16 units could be printed per batch to validate the reproducibility. The 3D-printed chewable gels were sealed in plastic bags and stored under ambient conditions (20–25 °C).

### 2.8. Visual, Sensory, Mass, and Dimensional Characterization of Chewable Gels

Evaluation of the chewable gels was carried out with a focus on children’s preferences, assessing appearance (color, opacity, and uniformity), odor (intensity and appeal), and consistency (durability during handling). The chewable gels were weighed with an analytical balance, and a box plot was created to analyze the weight variability. The thickness was measured with a caliper and compared to the virtual model.

### 2.9. Measurement of Water Activity

Samples of placebo and INH-containing 3D-printed chewable gels were tested for water activity. The test was adapted from [34], using a water activity meter (Lab Swift-aw, Novasina, Lachen, Zwitserland). The sample preparation involved cutting the formulation into pieces approximately 2 mm thick, which were then measured directly using the equipment. Each sample was tested in triplicate; analysis was performed on day one and after 35 days.

### 2.10. Texture Profile Analysis (TPA) of Chewable Gels

The chewing properties of the placebo and INH-containing 3D-printed chewable gels were tested using a TX-700 digital texture analyzer (Lamy Rheology, Champagne au Mont d’Or, France). Each sample was tested seven times. The device was equipped with a 10 kg load cell and a cylindrical probe with a diameter of 20 mm, simulating the chewing process of the teeth. The texture profile was analyzed using the TPA method and the TPA mode. The probe was lowered onto the sample at a controlled speed of 1000 mm·s^−1^, compressing it to 50% of the sample height. The initial force applied was 0.500 N. After compression, the probe was held in a waiting position of 10,000 mm for a delay time of 2 s, before being raised at a speed of 1000 mm·s^−1^. This process was repeated twice to obtain consistent data on the textural properties of the material.

Three basic parameters were determined based on the TPA curve: hardness, cohesiveness, and springiness. Hardness was defined as the first force peak (Fcmax1) on the TPA curve. Cohesiveness was the ratio of the force area during the second compression (A2) to that during the first compression (A1). Springiness was defined as the recovered sample deformation during the second compression. The maximum compression force was reached during the first compression (Fcmax1). The subsequent compression was performed with a lower deformation of the slightly recovered sample. Two further parameters were calculated from the estimated basic parameters: gumminess and chewiness. Gumminess is the product of hardness and cohesiveness, while chewiness is the product of gumminess and springiness [36].

### 2.11. Low-Vacuum Scanning Electron Microscopy (LVSEM)

Samples were analyzed in low-vacuum mode without conductive coating, using a Quattro ESEM microscope (Thermo Scientific, Waltham, MA, USA). The acceleration voltage was fixed at 5 kV with a vacuum of 10 Pa to minimize damage and ensure high-resolution imaging [37]. The method involved preparing the chewable gels by cutting them transversely and attaching them to a stub, ensuring both internal and external visualization at magnifications of 29×, 55×, 60×, and 153×.

### 2.12. Determination of the Isoniazid Content in the Chewable Gels

The content of INH in the chewable gels was quantified using high-performance liquid chromatography (HPLC), according to the method described in [38], as no method exists for chewable gels. Three units of chewable gels were dissolved in 200 mL of 0.1 mol L^−1^ phosphate buffer (pH 6.9) at 40 °C under magnetic stirring for 30 min, prepared in triplicate. After cooling to ambient conditions (20–25 °C), each solution was transferred to three different volumetric flasks, 25 mL of methanol was added, and the volume was completed with buffer solution. Placebo samples were prepared similarly to investigate the selectivity of the method. Chromatographic analysis was conducted with a UV detector (254 nm) and a C18 column (250 × 4.6 mm, 5 µm) (Shimadzu, Kyoto, Japan). The mobile phase consisted of 0.1 M phosphate buffer (pH 6.9): methanol (95:5, *v*/*v*) at a flow rate of 1.5 mL min^−1^. The medium of the samples was filtered through a 0.45 µm syringe membrane and the injection volume was 20 µL, with the column oven turned off and the ambient conditions maintained at 25 °C. Each sample was injected in duplicate. The relative standard deviation was required to be a maximum of 1.0%, while the column efficiency needed to achieve a minimum of 1800 theoretical plates was employed. The peak asymmetry was limited to a maximum of 2.0, and the retention factor was required to be at least 2.35. The response factor ratio between standard solutions was maintained within the range of 98% to 102%. Additionally, the relative standard deviation between sample solutions did not exceed 5.3%.

### 2.13. Determination of Degradation Products in the Chewable Gels

The determination of degradation products in the 3D-printed chewable gels was adapted from the determination of degradation products in the 3D-printed chewable gels was adapted from an internal methodology of Farmanguinhos/Fiocruz, as no specific method exists for chewable gels containing INH, and they were quantified using HPLC. Three units of the chewable gels were dissolved in 150 mL of 15 mM ammonium formate buffer (pH 7.0), at 40 °C, under magnetic stirring, for at least 30 min or until completely disintegrated, and they were prepared in triplicate. After cooling to ambient conditions (20–25 °C), the solution was transferred to a 200 mL volumetric flask, 20 mL of acetonitrile was added, and the volume was completed with diluent. A 10 mL volume was transferred to a 25 mL volumetric flask, and the volume was completed with diluent. Placebo samples were prepared similarly to investigate the selectivity of the method. Each sample was injected in duplicate.

### 2.14. In Vitro Drug Release Study

The drug release profile test was adapted from [38] for INH tablets. Dissolution tests were performed using a Varian VK 7010 automatic dissolution apparatus with a USP Type 2 paddle at 50 rpm. The dissolution medium was 900 mL of 10 mmol·L^−1^ hydrochloric acid at 37 ± 0.5 °C. Samples with a volume of 10 mL were collected at 5, 10, 15, 30, and 45 min, filtered through a 70 μm full-flow filter of Ultra-High-Molecular-Weight Polyethylene, and analyzed at 265 nm using a UV/Vis Spectrophotometer (UV/Vis, Shimadzu, Kyoto, Japan), using the medium to correct the background. The average dissolution rate was calculated, and results were compared with the results obtained from 100 mg commercial INH tablets.

### 2.15. Electronic Tongue—In Vitro Taste-Masking Test

A microfluidic impedimetric electronic tongue [39] composed of an array of four sensor units—one bare and three modified with 15 bilayers of PAH/PSS (poly(allylamine hydrochloride)/poly(sodium 4-styrenesulfonate)) nanostructured films [40], with two of them containing an additional 15 bilayers of PAH and silver or gold nanoparticles (PAH/AgNPs and PAH/AuNPs)—was utilized to evaluate the taste-masking property of 3DP chewable gels in salivary medium. Impedance data were collected with the impedance analyzer Solartron 1260A (AMETEK, Farnborough, UK) in the frequency range of 1 Hz to 1 MHz, applying an AC voltage of 25 mV at ambient conditions (20–25 °C). A syringe pump from New Era Pump Systems (New York, NY, USA) was used to inject liquid samples into the electrode channels at a flow rate of 15 mL/h. The electrical response of the array of four sensory units modified with organic/inorganic films was used to evaluate the taste characteristics of the formulation [41]. Impedance data were analyzed using Principal Component Analysis (PCA), a widely used statistical technique for dimensionality reduction and pattern exploration in complex datasets [42]. The dissolution medium used was an artificial saliva solution with a pH of 6.8 [43]. Chewable gels with (batches 1 and 2) and without INH (placebos) were sliced into 2 mm thick pieces and added to the artificial saliva at 40 °C, and were kept under stirring until complete dissolution. A 100 mg INH tablet was split into two equal parts, and one half was crushed as finely as possible, added to the saliva, and kept under agitation to simulate clinical practice. A physical mixture was prepared using powdered API of INH, maltitol syrup, and xylitol, and tested by dissolving it directly in the artificial saliva under magnetic stirring. Each sample was prepared in triplicate and tested seven times each.

### 2.16. Statistical Analysis

The results are expressed as the mean ± standard deviation (SD). Differences between the mean values of the control and experimental groups were assessed using Student’s *t*-test for independent samples. Statistical analyses were performed using Origin 9 software (OriginLab, Northampton, MA, USA. License nº PD6207E2F815604F).

## 3. Results

### 3.1. Preparation of the Intermediate Product Formulation

A total of 130 scientific publications were reviewed to guide the selection of polymers used as gelling agents in the formulations under study, based on their previous use in the preparation of chewable gels. From the initially listed excipients, those approved for oral use by the FDA and those that showed the best results among the 36 articles selected from the 130 initially listed were chosen for this work. The highest and lowest concentrations used in the articles were listed, and from there, the selection of excipients and concentrations was made.

Even though several formulations were not initially considered, the selection process followed the Quality by Test (QbT) approach, in which excipients and concentrations are rationally selected based on well-documented evidence, and then experimentally validated through internal reproducibility and performance metrics, such as gel homogeneity, process temperature, and ease of preparation. With the selected polymers and tested formulations, it was possible to evaluate the suitability of 3DP for chewable gel production. Different formulations were prepared using various combinations of hydrocolloids, pharmaceutical excipients, and sugar. All formulations were produced using a heated preparation process. After gelation, some formulations exhibited inadequate physical and mechanical properties for 3DP, such as high viscosity, lump formation, and stickiness. Among the hydrocolloids tested, gelatin and carrageenan gum were considered.

To prepare the chewable gels, the first polymer chosen was gelatin, a versatile and safe biopolymer derived from collagen. Gelatin is widely used in 3DP for pharmaceuticals and the food industries. It forms a three-dimensional network through hydrogen bonds when dissolved in hot water and cooled, providing good elasticity and strength in pharmaceutical chewable gels [44,45,46,47]. However, pure gelatin gels are inadequate for 3DP, due to their limitations in forming stable structures. To address this limitation, carrageenan, known for its gelling, stabilizing, and thickening properties, was combined with gelatin. The combination of gelatin and carrageenan forms strong, thermoreversible gels with excellent textural properties, making it suitable for the development of 3DP chewable gel technology [26,27,28]. Additionally, nutritive sweeteners such as xylitol and maltitol syrup were chosen for their low calories and good sweetness compared to table sugar [48,49]. The sweeteners applied are tooth-friendly and low-calorie, enhancing the overall quality and acceptability of the chewable gels [30].

Initially, the preparation process, which involved using open systems, such as beakers with heating plates, instead of closed systems, proved to be very challenging [50] because of significant water loss (evaporation) during manipulation, which affected the final concentration and the polymer incorporation [51]. Additionally, heterogeneous temperature distribution was observed in the plate heating process [52]. To address these issues, a closed system was adopted, minimizing water loss and ensuring better humidity and temperature control with thermal homogeneity, thus preventing premature polymer hydration and ensuring proper dispersion during optimization processes.

The final mean Brix value for the different batches was 65.2° ± 0.44° Brix, indicating consistent reproducibility across the samples. The soluble solids content obtained was lower than the typical range of 74–80° Brix reported in the literature for gelatin-based gummies [53]. The limitation observed in Brix values was due to the batch size and the minimum water required in the utilized system, which restrained an increase in the solids in the formulation. These optimizations will be addressed in future studies.

### 3.2. Rheological Analysis of the Intermediate Products

#### 3.2.1. Temperature Ramp and Shear Rate Sweep

**Figure 1:**
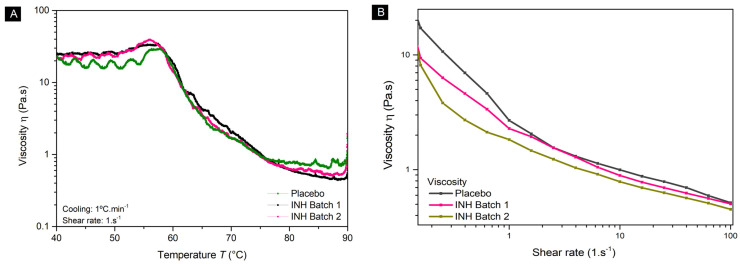
Rheological evaluation through (**A**) temperature ramp of 1 °C· min^−1^ and shear rate of 1 s^−1^, and (**B**) shear rate sweep from 0.1 to 100 s^−1^ at 70 °C.

The rheological behavior of the intermediate products was analyzed to evaluate their performance in 3DP. The results from the temperature ramp are shown in Figure 1A, and reveal that all formulations (placebo, Batch 1, and 2 with INH) retained structural integrity at ambient conditions (20–25 °C), but required a working range of 65–70 °C to achieve sufficient fluidity for extrusion while maintaining structural integrity. At temperatures above 75 °C, the viscosity dropped below 1 Pa·s, compromising print quality due to high fluidity, while a noisy behavior was obtained below 60 °C. The increasing viscosity behavior observed from 90 to 60 °C suggests exponential growth. However, a proper viscosity measurament could not be carried out from 60 to 40 °C, indicating the possibility of wall slipping phenomena due to the aprupt viscosity increase. Therefore, the temperature below 60 °C could not be safety evaluated for material extrusion [28,29]. Shear rate sweep tests confirmed pseudoplastic behavior, as shown in Figure 1B, with viscosity, a key property for extrusion, decreasing as the shear rate increased. Differences in initial viscosities were assigned to sample accommodation, but beyond 1 s^−1^, the materials behaved homogeneously. INH did not affect the rheological performance of the matrix, and pseudoplasticity aligned with the literature reports of the rate of carrageenan–polyol mixtures [28,29,33].

**Figure 2:**
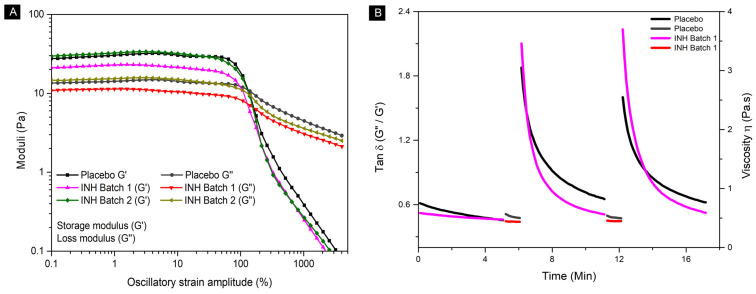
Rheological evaluation. (**A**) Strain amplitude sweep test at 70 °C, comparing placebo, batch 1, and batch 2. (**B**) Variation in tan δ and viscosity as a function of time for samples subjected to elastic recovery test at 70 °C, comparing placebo and INH batch 1.

Strain amplitude sweep and elastic recovery tests evaluated the intermediate products’ structural stability and viscoelasticity. The formulations with and without INH showed elastic behavior at low strain amplitudes (G′ > G″), transitioning to viscous behavior at higher strains, as shown in Figure 2A. The yield stress was equal to 18.97 ± 3.98 Pa, characterized as the onset of flow for the formulation at around this value, without significant differences between the INH and placebo batches [28,54,55]. Elastic recovery tests indicated that tan δ (G″/G′) increased to values larger than 1 during shear application, but decreased below this limit post shear, indicating effective restructuring and solidification. The results are shown in Figure 2B, respectively. This thixotropic behavior ensures that the material flows during extrusion and solidifies quickly after deposition, which is critical for maintaining the printed product’s shape [25,54]. These properties confirm the material’s suitability for 3DP applications.

#### 3.2.2. Simulation of Printing

**Figure 3:**
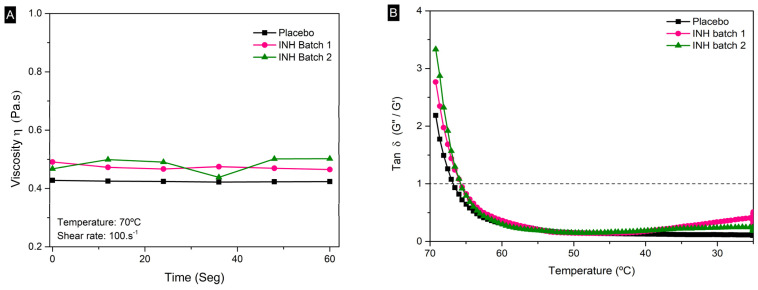
Rheological evaluation. (**A**) High shear rates to simulate extrusion. (**B**) Variation in tan δ as a function of temperature to simulate printing process and chewable gel formation.

High shear rates were applied to simulate extrusion, without any pattern alteration among the samples at this stage, as shown in Figure 3A. The process was followed by a cooling ramp from 70 to 25 °C, mimicking material deposition and layer solidification, as shown in Figure 3B. The results show a transition from viscous (tan δ ≥ 1′) to elastic behavior (tan δ < 1) as the material cooled, ensuring stable chewable gel formation. The thixotropic nature of the intermediate poducts facilitated quick structural recovery after shear cessation, ensuring precise layer deposition and structural integrity. The cooling phase supported uniform layer formation, which is critical for 3DP precision [28,54]. The results confirm that the intermediate products exhibit the necessary viscoelastic and thixotropic properties to enable successful 3DP [29,33,54]. The printing simulation validated the intermediate products’ performance under 3DP conditions.

### 3.3. Physical, Chemical, and Thermal Properties of Intermediate Products

**Figure 4:**
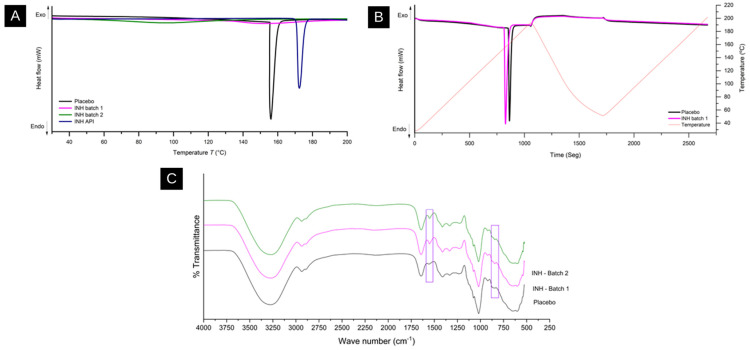
Physical, chemical, and thermal properties. (**A**) Variation in heat flow using DSC technique for placebo samples, batches 1 and 2 containing INH, and pure API. (**B**) Variation in heat flow analyzed using double-heating DSC technique for placebo samples and batch 1, containing INH. (**C**) FT-IR/ATR of placebo and INH batches 1 and 2 intermediate products. Rectangles in image highlight regions of FTIR spectrum where differences between samples are observed, but which do not prove significant interactions.

The chemical properties of INH in the intermediate products were investigated using DSC. The placebo formulations, batches 1 and 2 with INH, and the pure API were tested, as shown in Figure 4A. An endothermic peak between 170 and 178 °C was observed for the pure API [56]; however, the INH formulations showed no endothermic melting peak, indicating that the API was solubilized in the medium [57]. An endothermic peak in the placebo formulation between 155 and 162 °C suggests water loss from the polymer matrix. The double-heating technique was used to evaluate water removal, based on methods identifying and quantifying water in formulations [57,58,59]. Figure 4B shows similar thermal profiles for the placebo and INH-containing batches, differing from previous results. The endothermic event for the API batch occurred at around 155 °C, and for the placebo, it occurred at around 160 °C, with no further peaks indicating phase transitions. No thermal events near the pure API melting point were observed in the INH batch, confirming the absence of any insoluble particles in the gel. The reheated samples showed no further thermal events, indicating the absence of any degradation or contamination.

Infrared spectra of the placebo formulations and INH batches 1 and 2 were obtained; they show a significant overlap of the INH bands with those of the other components of the formulation. However, bands around 1600 cm^−1^, attributed to the deformation of the NH2 group of the hydrazide, can still be observed. The bands at 800 and 870 cm^−1^ indicate the angular deformation of the aromatic carbons of INH, as shown in Figure 4C [60,61]. The pronounced bands in the formulations containing the API demonstrate that the hydrazide structure was maintained after preparation, and no interactions were observed between the API and the formulation components. pH determination of the intermediate product formulations was carried out. Since the formulations did not require additional pH adjustments for polymer stabilization, extreme pH levels could compromise the polymer network and promote INH degradation. Considering that the pH of the API in aqueous solution ranges from 5.5 to 6.8, the values between 6.19 and 6.48 obtained for the formulations are within an adequate range to maintain INH stability [62].

### 3.4. Preparation and Characterization of 3D-Printed Chewable Gels

#### 3.4.1. Three-Dimensional Printing of Chewable Gels

**Figure 5:**
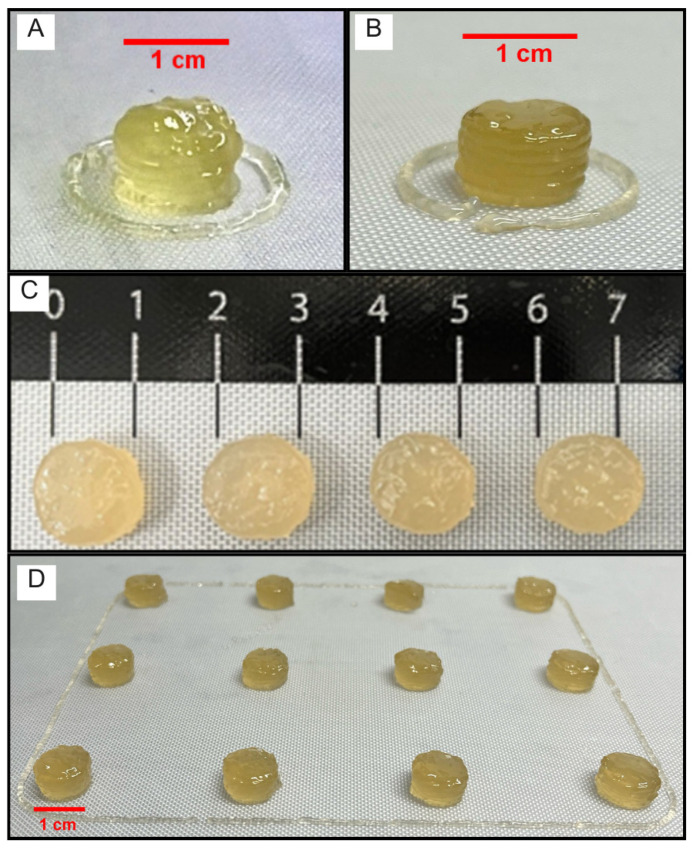
Optimization of 3DP chewable gel process. (**A**) Side view of chewable gel obtained by lower printing speeds, which led to filling failures and warping. (**B**) Side view of chewable gel with optimal conditions (speed: 10 mm/s, temperature: 68 °C, height: 5.6 mm, layer height: 0.8 mm). (**C**) Top view of optimized chewable gels, ensuring solid structure and reproducibility. (**D**) Multi-unit printing improved efficiency, while maintaining product reproducibility.

After the intermediate products were standardized, the chewable gels were printed to achieve a dose of 50 mg of INH. The selected model for 3DP preparation was a cylindrical shape, measuring 12 mm in diameter and 5.6 mm in height. This dosage selection was based on the use of half a tablet. Adjustments to printing parameters were made based on initial observations of defects. Initially, the printing speed was adjusted to 8 mm s^−1^, and then increased to 10 mm·s^−1^ and higher. Slower speeds allowed more time for material deposition, but resulted in filling failures and deformations due to excess material deposition (Figure 5A). Speeds above 10 mm s^−1^ compromised layer precision, producing a thinner but consistent filament that did not yield the desired product. Fixing the speed at 10 mm·s^−1^ proved ideal for balancing material deposition and process efficiency. According to Mushtaq et al. [63], the optimization of printing speed and fill density in 3DP demonstrated that the printing speed affects the fill precision and surface quality of the printed material.

Building on the adjustments made to the printing speed, the temperature settings were also crucial for achieving optimal results. The initial temperature of 70 °C, studied in the rheological tests, caused the deformation of the tablets after printing. As shown in the rheological tests, after printing, the sample needs to be restructured quickly to recover its solid behavior and preserve the desired shape. Moreover, this dynamic behavior will depend on the rate of heat transfer when the sample is submitted to the environment temperature, and also on the initial degree of sample structuring at the chosen temperature. Therefore, as a solution, a lower temperature of 68 °C provided better layer adhesion and a solid structure. This means that the initial structure degree was increased, due to the larger storage modulus and yield stress at lower temperatures. However, the general rheological behavior of the sample at 68 °C should remain similar to that at 70 °C. Lower temperatures were also tested, but resulted in clogs and increased pressure requirements. Incorrect or excessively high temperatures can cause excessive melting or insufficient melting of any polymer, leading to adhesion failures and product defects [64]. The temperature adjustment ensured that the material was adequately melted for extrusion, while maintaining the integrity of the printed layers, ultimately leading to a more reliable and consistent product.

Following the adjustment of printing temperature, the layer height, fill pattern, and extrusion control were also optimized. A nozzle with an output diameter of 1.2 mm was chosen for all tests. The chewable gel height was set at 5.6 mm, and the layer height was standardized at 0.8 mm, after smaller heights resulted in precision failures and nozzle clogs. A rectilinear fill pattern was chosen, and fill densities of 40% ±10% were tested. A 30% density did not adequately fill the tablet, making it friable and difficult to handle, while a 50% density overfilled it, causing excess material. Therefore, a 40% fill density was selected to balance tablet solidity and desired flexibility, achieving excellent unit standardization (Figure 5B,C). During syringe cartridge changes, minor precision losses in extruded material occurred, highlighting the printer’s difficulty in maintaining constant pressure on the plunger. Studies have emphasized that layer height and fill density directly affect the integrity and flexibility of the finished product, showing that fine adjustments during extrusion and printing improve filament stability [65]. It was possible to observe an evident difference in the final size of the chewable gel, despite no significant rheological changes between them or in the printing process.

After optimizing the layer height, fill pattern, and extrusion control, the process optimization and the number of printed units were addressed. Initially, the 3DP process results were achieved with a single printed unit at a time. As printing parameters were adjusted and reproducibility was achieved, the number of units printed simultaneously was gradually increased. The printing was performed with 2, 4, 9, and 16 chewable gels at a time, with a new tablet printed only after the previous one had been completed (Figure 5D). The ability to print multiple units increased the process efficiency by reducing the total operation and handling time, while maintaining the necessary product characteristics. The production of 16 units maintained a time of 80 s per unit, as speed parameters were not altered. This optimization allowed for automated manufacturing of multiple units, although some variation in individual unit weight was observed, without affecting the average weight, as will be shown in the next section. The robustness and reproducibility of the process are supported by previous studies demonstrating the uniformity and precision of 3DP for solid pharmaceutical forms, even in different environments [66]. Additionally, large-scale production of pharmaceutical forms via 3DP is reliable and scalable, indicating the maturity of the technology for future applications [67].

#### 3.4.2. Visual, Sensory, Mass, and Dimensional Characterization of Chewable Gels

The visual evaluation after printing multiple units of chewable gels revealed satisfactory properties for a chewable gel, such as uniform edges, a homogeneous yellowish color, and good layer adhesion with minimal print material residue, indicating a consistent production process. Additionally, the layer adhesion was adequate, with no evidence of separation or fragmentation when handled gently. After printing, the chewable gels exhibited the characteristic strawberry flavor. The main concern was the observation of slight syneresis around the units immediately after printing, which stabilized and did not affect the overall appearance of the pharmaceutical form. Syneresis in carrageenan gels is the spontaneous expulsion of water or other liquids when the gel is at rest. This occurs due to the polymer network’s reorganization pushing water to the surface, which can be attenuated by incorporating other polymers, such as gelatin [68,69].

**Figure 6:**
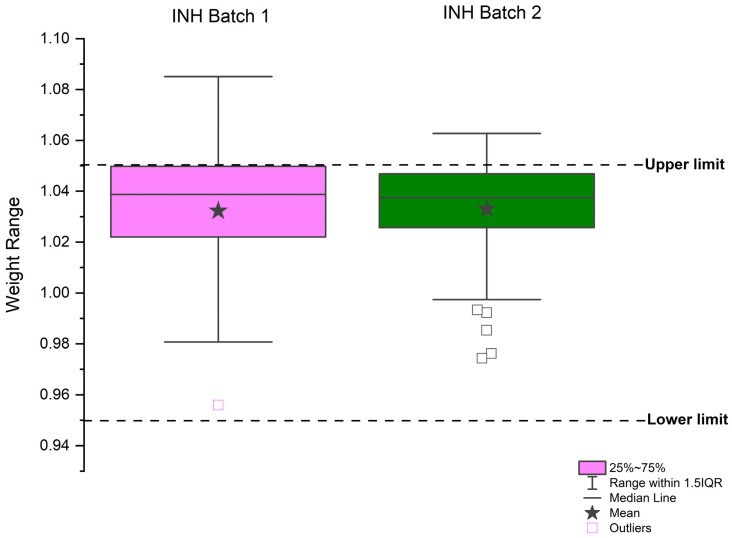
The weight variability of the produced chewable gels. The weight results after printing the batches are shown. For batch 1, the average weight was 1.0320 g and the median was 1.0388 g. For batch 2, the average weight was 1.0331 g and the median was 1.0373 g.

The main objective of characterizing the chewable gels was to evaluate the conformity of the produced samples with pharmacopeial parameters and those established during development. The USP specifies that tablets with an average weight of 250 mg or more can vary up to 5% from the declared average weight [70]. Considering that the chewable gels produced in this work had a nominal weight of 1.00 g, the individual unit weight was expected to be between 0.95 g and 1.05 g. The weight results after printing the batches are shown in Figure 6. For batch 1, the average weight was 1.0320 g and the median was 1.0388 g. For batch 2, the average weight was 1.0331 g and the median was 1.0373 g. Both the averages and medians are in accordance with pharmacopeial specifications; however, the dispersion in individual weights reveals variability that could impact the uniformity of the tablets. This level of dispersion suggests that the 3DP process must be improved to scale up production. Adjustments can be made through an experimental design matrix, adjusting in-process control parameters to improve consistency among chewable gel units, which will be addressed in future work [71,72].

After the weight evaluation, the dimensions of the chewable gels were measured 15 min post printing. The virtual design dimensions were 12 mm in width and 5.6 mm in height. The dimensions are specified in Table 3. The height may exceed the expected value due to a rheological phenomenon known as “extrudate swell”, which occurs when the cross-section of the filament increases in size after exiting the die, in response to the fluid’s elasticity. This effect is pronounced in polymeric fluids, where the extrudate diameter can exceed the die diameter by up to 300%, depending on the material’s elasticity [73].

#### 3.4.3. Evaluation of Water Activity and Isoniazid and Degradation Product Contents in Chewable Gels

Water activity (Aw) represents the free water available to interact with formulation components and the environment, influencing chemical degradation, microbial growth, and physical changes. In solid and semi-solid formulations like chewable gels, high Aw values can accelerate reactions that compromise INH stability [31]. Gummy candies with high soluble solids and polymer contents demonstrate excellent microbiological stability due to matrix density, eliminating the need for preservatives. Controlled packaging and storage are crucial to maintain product integrity [74,75]. The results showed stable Aw values for the three formulations over 35 days, with a downward trend, but no significant differences observed. The initial water activity (Aw) values on Day 1 for INH batches 1 and 2 ranged between 0.784 and 0.793, whereas those for the placebo ranged from 0.777 to 0.780. By Day 35, INH batches 1 and 2 showed 0.689 to 0.761, while the placebo recorded values between 0.755 and 0.761. The standard deviation for the INH batches was higher on Day 1 (±0.027), and decreased by Day 35 (±0.003), indicating greater stability over time. In contrast, the placebo showed low variation from the beginning (±0.003 on Day 1 and ±0.002 on Day 35), suggesting uniform behavior and the need for formulation optimization to minimize free water. Increasing the solids content could help to maintain Aw specifications [76].

**Figure 7:**
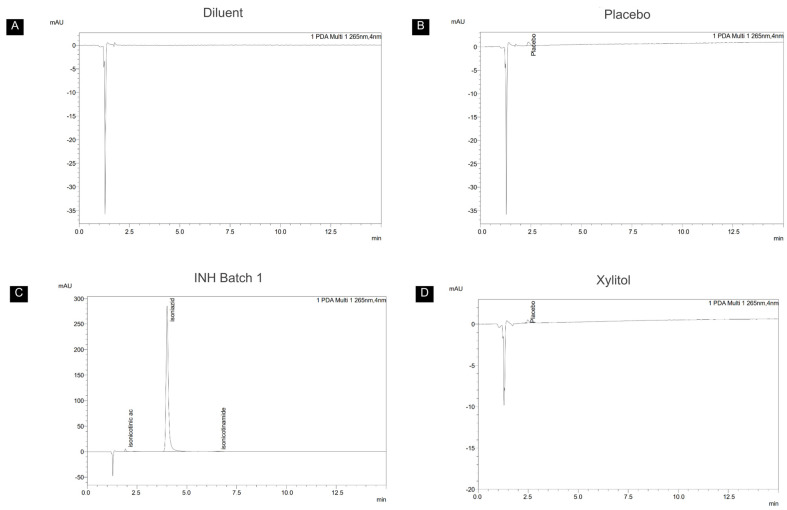
Representative chromatograms of degradation product analysis. Chromatograms of (**A**) diluent, (**B**) placebo, (**C**) INH batch 1, and (**D**) xylitol.

The evaluation of the chewable gels showed an average content of 103.4 ± 1.16%, in accordance with pharmacopeial specifications [70]. Batches 1 and 2 contained 51.7 ± 0.70 mg and 51.7 ± 0.47 mg of INH per unit (n = 18), respectively, both within the specified range of 45.0–55.0 mg per tablet and 90.0–110.0% of the declared amount. The total impurities were 1.2% in batch 1 and 1.3% in batch 2. Figure 7 shows the chromatograms obtained, with the two degradation products identified, isonicotinic acid and isonicotinamide. Degradation processes like deamination, which involves the cleavage of the bond between the carbonyl group (C=O) and the terminal amino group (-NH_2_), contribute to the formation of these impurities [77]. This reaction is energetically favorable and accelerated by factors such as temperature and humidity, leading to the formation of more stable products, like isonicotinic acid and isonicotinamide [78,79].

#### 3.4.4. Evaluation of Texture Profile Analysis

**Figure 8:**
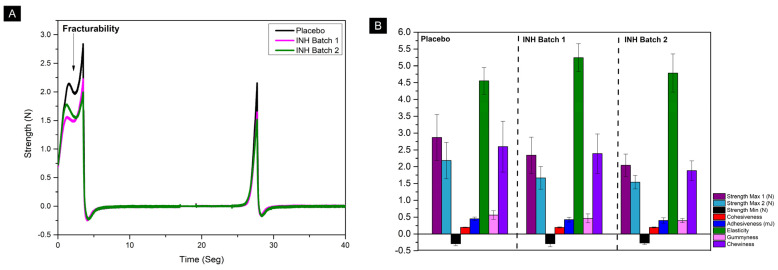
Texture profile analysis. (**A**) Graph of applied force and sample deformation recorded over time during texture profile analysis (n = 7). (**B**) Secondary parameters of texture profile analysis.

During the TPA, the applied force and sample deformation were recorded over time, resulting in a force–deformation curve. Figure 8A shows the average values (n = 7) for the chewable gel samples. The samples exhibited good reproducibility, with no abrupt differences between the tests. The first compression shows a wave-like behavior related to the material’s fracturability, demonstrating a fracture tendency before reaching the first peak of maximum hardness force [80]. The hardness, indicated by Maximum Force 1, appeared to be higher in the placebo sample than in the INH sample gels. Secondary parameters were determined from the recorded values, as shown in Figure 8B and Table 4. The secondary TPA parameters showed no differences between the samples, as the values showed no significant differences (*p* > 0.05) among them. This indicates texture reproducibility, regardless of the presence of INH in the formulation.

Comparing the results obtained with the experiments conducted on commercial vitamin chewable gels in ref. [81], which proposes quality parameter specifications, it is possible to identify correlations and indicate potential improvements. The average hardness of the placebo tablets was slightly higher than that of the drug-containing samples, indicating that the placebo was more resistant to compression. However, both formulations showed hardness values below the average hardness range observed in commercial chewable gels. For children, a lower hardness value is preferable, as it could facilitate the chewing process and INH release. Although a softer-chewing gum could pose a challenge during transportation, this is not a major issue, as it is intended for extemporaneous use and can be individually packaged and stored in designed on-demand packaging to protect it. The adhesiveness of the three formulation batches was similar, but higher than that of commercial gummies, which could result in a sticky sensation and decrease acceptance. Some adjustments in the formulation could reduce adhesiveness, such as coating the tablet surface with crystalline material. The cohesiveness was relatively low for both the placebo and drug-containing batches compared to commercial ones, indicating that the formulated chewable gels tend to break down more easily in the mouth. For children, low cohesiveness facilitates the breakdown and chewing of the gummy, making consumption safer by generating smaller pieces that could prevent choking [81].

Zhu et al. [82] highlighted that increasing the gelatin concentration in gummy formulations reduces adhesiveness and increases cohesiveness, due to greater molecular interaction. Conversely, the addition of carrageenan hinders this interaction, increasing adhesiveness and reducing cohesiveness. Higher gelatin concentration also increases chewiness and elasticity. Balancing these components is essential to maintain textural properties and acceptance by children. The low thermal stability of gelatin limits its application in 3DP, especially at temperatures above 40 °C, where it transitions from solid to liquid, affecting shape retention after printing. Parameters like hardness and gumminess directly impact the printing process: high values can lead to issues like poor extrusion and nozzle clogging, while very low values hinder shape retention. Qiu et al. [83] also demonstrated that reducing cohesiveness and elasticity improves printing precision. Therefore, the development of suitable formulations for 3DP must consider how component interactions affect textural properties to enhance the patient experience and printing performance. While this work achieved a balance between printing parameters and shape maintenance, adjustments to provide improvements with respect to the TPA consumption preferences can compromise the printing process.

#### 3.4.5. Low-Vaccum Scanning Electron Microscopy

**Figure 9:**
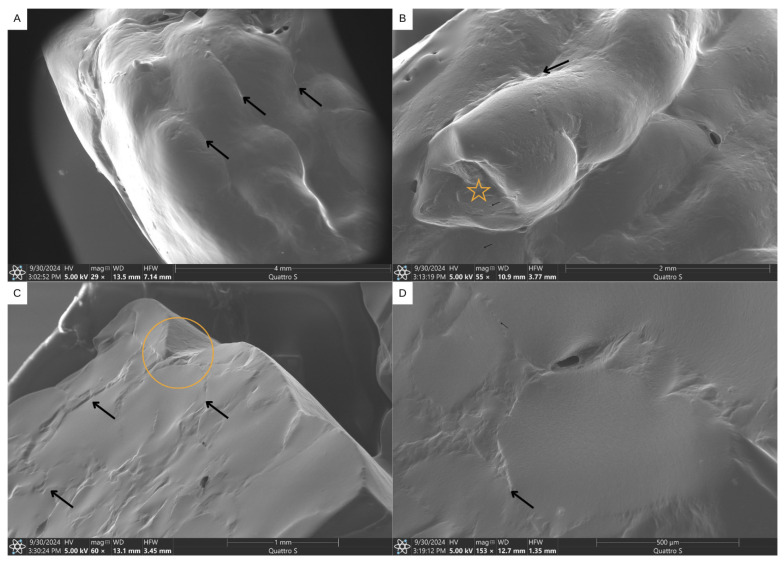
Low-vacuum scanning electron microscopy of INH chewable gels. The arrows in the images indicate the division between filaments or layers formed during the printing process. (**A**) Lateral (with layer indication) and top surfaces. Mag 29×. (**B**) A lateral surface with layer and filament indication (the star represents the end of printing, where the filament breaks). Mag 55×. (**C**) An internal surface with indication of filament division and the gap between the external lateral surface layer, indicated with a circle. Mag 60×. (**D**) An enlarged internal surface, with indication of filament ends. Mag 153×.

Microscopic analysis of the tablets was conducted using the LVSEM method to observe the characteristics of the lateral and internal surfaces of the samples. In the images (Figure 9), it was possible to observe that the lateral surface of the tablets presented well-defined filaments with good adhesion between them, and it was possible to see the division between filaments or layers formed during the printing process. However, the surface of the filaments was not homogeneous (Figure 9A,B); nevertheless, no agglomerates were observed [84]. The internal surface, on the other hand, presented distinct characteristics. During the internal filling of the layers, fusion occurred between them, resulting in a smooth and continuous surface, with light distinction of the filaments that made up the internal structure (Figure 9C,D). The definition of the filaments on the lateral surface was good, but homogeneity of the layer thickness was not observed. The internal surface demonstrated fusion between the filaments and layers, resulting in a smoother surface. This observation indicates that good fusion between the printing filaments results in chewable gels that are dense in structure and have a good appearance [21].

#### 3.4.6. In Vitro Drug Release Study

**Figure 10:**
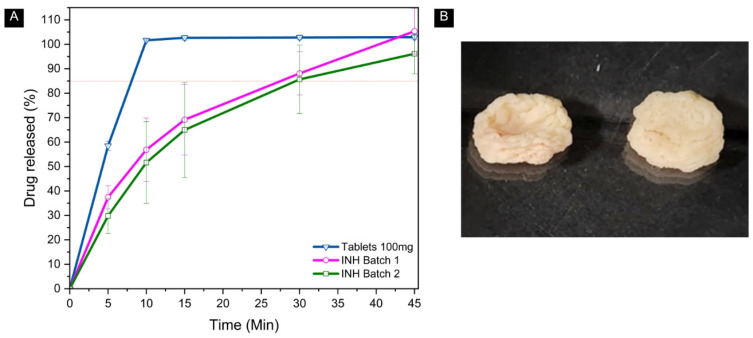
A comparative dissolution profile between traditional tablets and 3D-printed chewable gels. (**A**) The percentage dissolved is greater than 85% in 30 min. The red dashed line represents 85% of the API released. (**B**) The matrix of the chewable gels after the experiment time.

The dissolution methods described in the USP offer different approaches for evaluating the release of active ingredients. The method for dietary supplements (<2040>) focuses on dietary ingredients, ensuring that at least 75% of the labeled content is released within the specified time. However, this method is not specific to medications, which require precise release data to achieve a therapeutic window. The USP <724> Drug Release method determines compliance with drug release requirements specified in individual monographs [70]. For the chewable gels intended for immediate release, the dissolution profile was evaluated based on the INH monograph in the USP for INH tablets, using 0.01N hydrochloric acid as the dissolution medium [38]. According to USP <1090> Assessment of Drug Product Performance—Bioavailability, Bioequivalence, and Dissolution, immediate-release drugs should dissolve at least 85% of the API within 30 min to ensure proper bioavailability and therapeutic efficacy [70]. The INH chewable gel samples from batches 1 and 2 achieved drug release of 105 ± 9,4% and 96 ± 8,2%, respectively, at the end of 45 min, with more than 85% released within 30 min. The reference tablets showed 100% release in 10 min, reaching 103 ± 1.04% after 45 min, as shown in Figure 10A. Despite initial deviations, both formulations met the immediate-release objective. INH is considered a candidate for bio-waiver due to its high solubility and permeability, allowing registration without in vivo studies, using only in vitro dissolution tests [85]. The dissolution profiles of the two INH chewable gel formulations were compared to the reference tablet using the similarity factor (F2). The calculated values were 27 for batch 1 and 25 for batch 2, both below the acceptance criterion of 50, indicating that neither formulation exhibited dissolution behavior equivalent to that of the reference. However, as more than 85% of the API was released within 30 min, both chewable gels meet the criteria for immediate-release drug products according to USP [70]. This was true even without cutting the gels, according to the dissolution criteria (Figure 10B). This indicates that even if a child accidentally swallows the tablet while taking it, the medication release is not affected, with the entire structure of the gel being maintained, as observed by Liang et al. [50].

#### 3.4.7. Electronic Tongue—In Vitro Taste-Masking Test Evaluation

**Figure 11:**
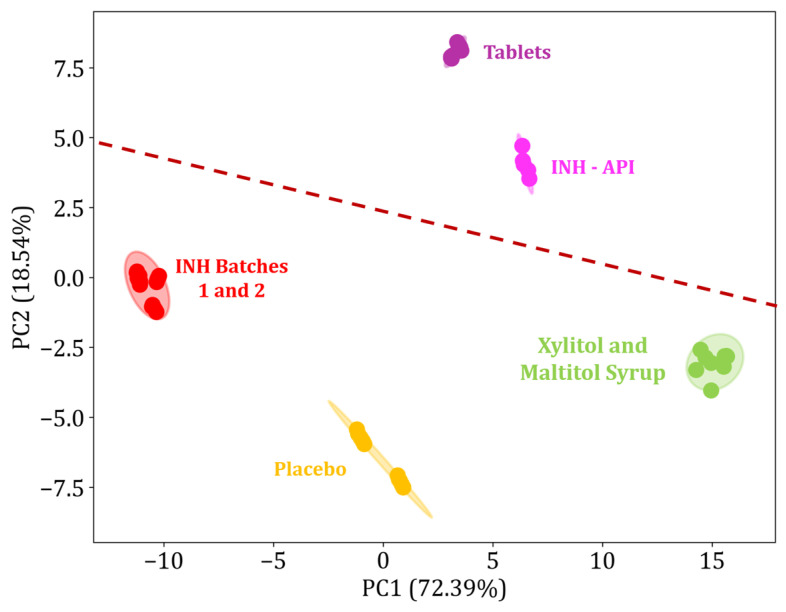
A PCA plot for the placebo and INH chewable tablets (mg/mL), 100 mg isoniazid tablet (Halved), INH IFA powder, maltitol syrup, and xylitol. The dashed line represents a separation trend observed in the PCA plot, highlighting differences in taste profiles among the tested samples.

A taste-masking assessment of the chewable gels was conducted using an impedimetric electronic tongue. The in vitro experiment was conducted with the INH samples (batches 1 and 2), the placebo, a commercial INH tablet split in half (pediatric dosage), the isolated API, and excipients of the tablets corresponding to the sweeteners maltitol syrup and xylitol. The PCA plot is presented in Figure 11, with the samples coded by different colors. A total of 91% information from impedance experiments, which indicates high data correlation, is represented by the two first principal components. The distance between the replicas within the same group indicates the variability and homogeneity of the samples, while the distance between different groups suggests the degree of similarity in the taste profile. Sweeteners such as maltitol syrup and xylitol have a similar taste to sugar, characterized as pleasant to the palate [48,86]; this group is close to the placebo and INH batches, indicating similarity in taste. On the other hand, the API is considered a reference for a bitter and unpleasant taste [87,88]; note that it is located close to the split commercial INH tablet, reinforcing the fact that splitting the tablet impairs the palatability of the medication. The results show that the chewable gels containing the INH significantly deviate from both references (sweeteners and API), suggesting a balanced taste. These results highlight the importance of developing suitable formulations, not only in terms of therapeutic efficacy, but also to improve patient acceptability. Overall, from the in vitro palatability test conducted, the palatability of the chewable gels containing INH is promising for taste-masking, corroborating the literature on the application of the 3DP SSE technique in the production of chewable gels for this purpose [89].

## 4. Conclusions

This study utilized SSE-3DP technology to develop personalized chewable gels containing INH for pediatric LTBI treatment. The formulation of IPs became homogeneous, through the absence of uncontrolled water loss by evaporation. The batches produced using the closed system showed no variations during preparation or in their final appearance. The rheological properties of the optimized formulations showed promising results capable of ensuring structural integrity and fluidity during the printing process. Following the optimization of the tested printing parameters, the process proved to be reproducible, enabling the automated manufacturing of multiple units simultaneously. The 3DP chewable gels exhibited consistent quality, with uniform weight and dimensions, stable AW, and texture properties suitable for children. The contents of INH and degradation products in the chewable gels were in accordance with pharmacopeial specifications, and the in vitro drug release profile met immediate-release criteria, with over 85% of the drug released within 30 min. The taste-masking evaluation demonstrated enhanced palatability relative to traditional INH tablets, indicating a balanced taste profile. These findings underscore the potential of SSE-3DP technology to personalized dosage forms that meet regulatory standards for immediate-release tablets and enhance patient experience.

The reproducibility and flexibility of SSE-3DP technology make it a promising solution for producing small-batch, personalized medications. This technology can be deployed in remote and hard-to-reach areas, improving the accessibility and effectiveness of pediatric LTBI treatment. Future research should focus on further optimizing the printing process, assessing long-term stability, and conducting clinical trials to validate the efficacy and acceptability of these chewable gels in pediatric populations. Additionally, studies on formulation stability and drug crystal structure will support the advancement of tailored pediatric medicines. With appropriate control strategies and the development of online quality-control systems, 3D printing technology is poised to significantly enhance personalized medicine, supported by continuous improvements in relevant regulations led by authorities.

## Figures and Tables

**Table 1:** Composition of intermediate products.

Materials	Placebo (%)	INH (%)
INH	-	5.00
Carrageenan gum	1.00	1.00
Gelatin	3.00	3.00
Xylitol 1 ^a^	3.00	3.00
Xylitol 2	23.50	18.50
Maltitol syrup	46.50	46.50
Strawberry flavor	0.50	0.50
Purified water	22.50	22.50

^a^ The amount of xylitol was divided into two parts to avoid accelerated hydration of the polymers in the formulation, which prevents hydration inside the particles and favors lump formation [32].

**Table 2:** Optimized printing parameters adjusted on 3D printer.

Parameter	Value
Model	Cylinder (selected STL file)
Speed	10 mm·s^−1^
Virtual size	12 × 12 × 5.6 mm
Nozzle diameter	1.2 mm
Layer height	0.8 mm
Number of layers	7
Fill density	40%
Fill pattern	Rectilinear

**Table 3:** Dimensions of chewable gels. SD—standard deviation.

Batch	Width Average (mm)	Width SD (mm)	Height Average (mm)	Height SD (mm)
INH batch 1	11.22	±0.51	6.62	±0.43
INH batch 2	11.39	±0.37	6.64	±0.28

**Table 4:** Mean values of secondary parameters of texture profile analysis.

Parameter	Placebo	INH Batches
Hardness (N)	2.870 ± 0.68	2.200 ± 0.46
Adhesiveness (mJ)	0.426 ± 0.06	0.426 ± 0.06
Cohesiveness	0.196 ± 0.01	0.195 ± 0.01
Elasticity	4.554 ± 0.39	5.029 ± 0.53
Chewiness	2.595 ± 0.75	2.153 ± 0.52

## Data Availability

The data are contained within the article.

## Supplementary Materials

The following supporting information can be downloaded at: https://www.mdpi.com/article/10.3390/pharmaceutics17050658/s1, Video S1—Demonstration of the chewable gel printing process.

## Abbreviations

The following abbreviations are used in this manuscript:

3DP	Three-dimensional printing
SSE	Semi-solid extrusion
TB	Tuberculosis
LTBI	Latent tuberculosis infection
INH	Isoniazida
LVR	Linear viscoelastic range
DSC	Differential scanning calorimetry
PCA	Principal Component Analysis
HPLC	High-performance liquid chromatography
AW	Water activity
LVSEM	Low-vacuum scanning electron microscopy
USP	United States Pharmacopeia
TPA	Texture profile analysis
UV	Ultraviolet rays
UV/VIS	Ultraviolet visible spectroscopy
API	Active pharmaceutical ingredient
SD	Standard deviation
CAD	Computer-Aided Design
ATR/FTIR	Attenuated total reflectance Fourier-transform infrared
STL	Standard Tessellation Language
G′	Storage modulus
G″	Loss modulus
